# Personalized outcomes for hip and knee replacement: the patients point of view

**DOI:** 10.1186/s41687-021-00393-z

**Published:** 2021-11-04

**Authors:** Robin R. Whitebird, Leif I. Solberg, Jeanette Y. Ziegenfuss, Stephen E. Asche, Christine K. Norton, Marc F. Swiontkowski, Steven P. Dehmer, Elizabeth S. Grossman

**Affiliations:** 1 Morrison Family College of Health, School of Social Work, University of St. Thomas, 2115 Summit Ave, Office: SCB #106, St. Paul, MN 55105 USA; 2 HealthPartners Institute, Minneapolis, MN USA; 3 Patient Advocate and Independent Research Consultant, Cottage Grove, MN USA; 4 Department of Orthopeadic Surgery, University of Minnesota Medical School, Minneapolis, MN USA

**Keywords:** Patient reported outcomes (PRO), Performance measurement, Orthopedic surgery, Clinical care, Patient perspective, Shared decision-making, Patient engagement, Patient-centered care

## Abstract

**Background:**

Patient reported outcome measures (PROMs) are increasingly being incorporated into clinical and surgical care for assessing outcomes. This study examined outcomes important to patients in their decision to have hip or knee replacement surgery, their perspectives on PROMs and shared decision-making, and factors they considered important for postoperative care.

**Methods:**

A cross-sectional study employing survey methods with a stratified random sample of adult orthopedic patients who were scheduled for or recently had hip or knee replacement surgery.

**Results:**

In a representative sample of 226 respondents, patients identified personalized outcomes important to them that they wanted from their surgery including the ability to walk without pain/discomfort, pain relief, and returning to an active lifestyle. They preferred a personalized outcome (54%) that they identified, compared to a PROM score, for tracking progress in their care and thought it important that their surgeon know their personal outcomes (63%). Patients also wanted to engage in shared decision-making (79%) about their post-surgical care and identified personal factors important to their aftercare, such as living alone and caring for pets.

**Conclusions:**

Patients identified unique personalized outcomes they desired from their care and that they wanted their orthopedic surgeons to know about. Asking patients to identify their personalized outcomes could add value for both patients and surgeons in clinical care, facilitating more robust patient involvement in shared decision-making.

## Key points

1. Patients can identify personalized outcomes for their care that they want their surgeons to know about.
2. Most patients prefer to use a personal outcome they identify rather than a PROM score for tracking progress in their care.
3. Adding personal outcomes identified by a patient to PROMs information may facilitate more robust patient–clinician interactions about their care.

## Introduction

Patient reported outcome measures (PROMs) have received increased attention for care improvement, including in orthopedics [1–3]. This attention aligns with the evolving priority to emphasize a more patient-centered approach in care that improves patient satisfaction and experience, fosters shared decision-making, and improves the quality and value of health care [4, 5]. Patient-reported outcomes, defined as reports by a patient about their health, form the structure of PROMs and are primarily collected through multi-item questionnaires and reported as summary scores [6]. PROMs focus on symptoms, function, and health-related quality-of-life questions, with various PROMs developed to address particular medical diagnoses, procedures, and/or conditions [3, 7]. Whether PROMs should also provide a patient’s perspective regarding the treatment or desired outcomes of the care they receive has thus far not been a major consideration in their development, selection, and use.

PROMs originated in research for the purpose of assessing effectiveness of medical and surgical treatments. Their use has expanded to measure health care quality for reimbursement, and more recently focuses on care improvement, performance measurement, and comparison feedback to clinics and clinicians [1, 7–9]. There are a wide variety of PROMs available, with 42 unique PROMs in use for total joint arthroplasty alone [2, 10]. Concerns have been raised about the selection and use of PROMs for clinical and surgical care including selection of measures, validity and reliability, and the importance of involving patients in PROM development [3, 4, 11–15]. Of particular importance is how PROMs can be incorporated into the clinical care of individual patients and whether they are perceived as useful and meaningful to both providers and patients [8, 16, 17]. Prior research indicates that while providers may benefit from PROMs use, patients can be confused about their purpose and meaning [18]. PROMs also focus on patient’s perceived health status and functioning but have not reflected what patients individually desire in their care outcomes. Research has been conducted examining patient preferences and expectations about their care experiences [19], but what they desire as outcomes of surgical care can be quite different.

Aligning treatment and outcomes with what patients perceive as most meaningful and important in their care and its outcomes, defined as their personal outcomes, could be key to good clinical outcomes and affect patient satisfaction [20]. We examined the use and value of PROMs in a clinical orthopedic setting that developed a systematic approach to collecting and integrating PROMs into patient care. The purpose of this study was threefold: (1) to learn from patients what outcomes are most important to them in their decision to have hip or knee replacement surgery; (2) to investigate the perspective of patients regarding use of PROM scores for their clinical care in comparison to their desired individualized outcomes and priorities, and their perspectives on shared decision-making; and (3) to identify factors important for aftercare following surgery.

## Materials and methods

This cross-sectional survey study is part of PROMOTE: Patient Reported Outcomes Measure Optimization through Technology and Engagement, funded by the Agency for Healthcare Research and Quality and conducted in the upper Midwest at a nonprofit research center affiliated with an integrated, multi-specialty health system including 50 clinics, 6 hospitals, and care for 1.2 million patients. The PROMOTE Study is focused on improving collection and use of PROMs in clinical care and quality improvement. The study survey collected data from patients seen in orthopedics for hip or knee replacement surgery and was conducted from November 2018 to January 2019.

### Participants and recruitment

**Fig. 1:**
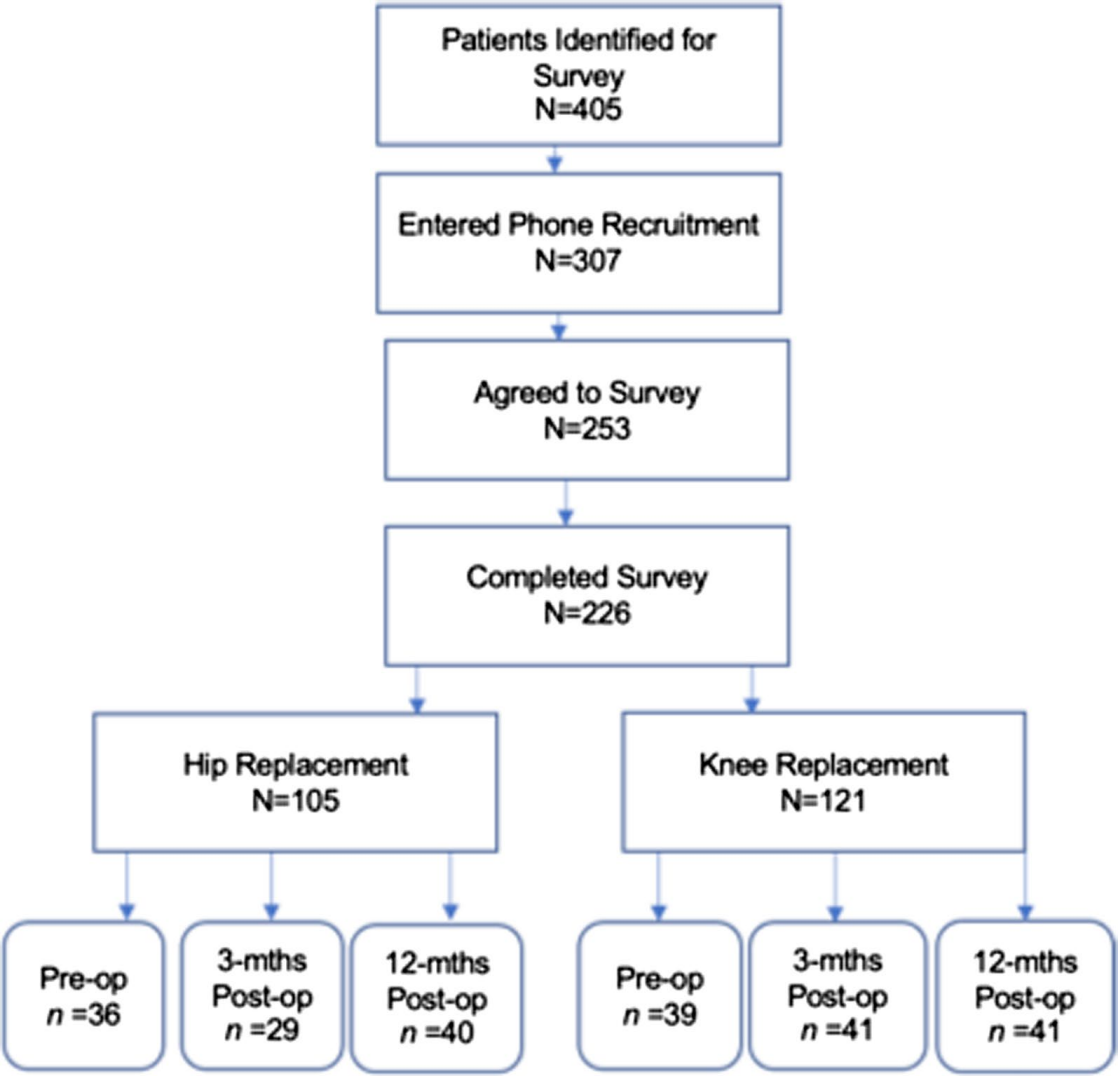
Flow of survey respondents

Study participants were a stratified random sample of adult orthopedic patients who were eligible to participate if they had scheduled or recently experienced a hip or knee replacement surgery and completed a PROMs survey, which they routinely receive at 3 time points—pre-operatively, and 3- and 12-months post-operatively. Potential participants were identified using PROMs administrative data and then stratified based on surgery type (hip/knee) and time period (pre-operative, and 3- and 12-months post-operative). An approximately equal number of patients were selected for each of the resulting six strata. A total of 405 patients were identified for survey, which was designed for online administration matching the PROMs system. Initial contact was made via telephone to secure consent to use patient email for the survey participation. There were 307 patients successfully contacted by telephone (94 could not be reached following multiple attempts); 253 of these agreed to receive the email survey or requested a paper survey and 226 participants completed the survey. The survey response rate was 56% with a survey participation rate of 74% (completed surveys/phone recruitment contact). Respondents included 105 patients with hip replacement and 121 with knee replacement (Fig. 1).

Eligible participants were mailed a letter informing them about the survey and that they would be contacted by phone regarding participation. This was followed by phone contact from a survey research center professional interviewer who determined willingness to participate and obtained consent for survey participation and email addresses for web-based survey participation; a series of up to three email reminders and four additional phone call reminders were made to individuals that agreed to complete the survey but did not complete upon initial email contact. Interested participants who requested a mail survey were sent one instead of the online survey; of the 226 completed surveys, 218 were completed online and 8 by mail. Patients received a $10 gift card as a thank-you for survey completion. The study was reviewed, approved, and monitored by the local Institutional Review Board.

### Data collection

The study questionnaire was developed by the study team based on prior interviews conducted with a group of 65 patients having hip or knee replacement or spine surgery from an earlier phase of the PROMOTE study [18]. For this study we looked at confirming results focusing only on hip and knee replacement patients. Interviews in the prior study explored the most important outcomes patients wanted from their surgery, as well as factors about their own life they thought were important to their postoperative care. We used results of that study to develop a structured questionnaire designed for hip and knee replacement patients. The questionnaire contained 18 questions (tailored for the time point relative to surgery) addressing: personalized outcomes patients identified as most important to them for their surgery, their interactions with their surgeons about their personal outcomes, their perceptions of shared decision-making and whether their surgery was a success, their perception of PROMs used in their clinical care, and personal factors they identified as important that might affect their care following surgery. Personal outcomes, defined as specific outcomes that patients identify as important to them, and personal factors were identified by presenting pre-specified lists of 17 and 16 items respectively that covered most responses from the interviews, as well as an opportunity to provide “other” choices. For personal outcomes, respondents were asked to pick the three most important outcomes for them in their surgery and care. The order of pre-specified outcomes was randomized to one of 8 survey versions to reduce order effects. For personal factors important to their aftercare, they were asked to check all that apply and to add others not included.

### Data analysis

Individual survey items were summarized with descriptive statistics (frequency, percentage, mean, standard deviation) within the knee and hip replacement patients separately and in total. Differences in items by type of surgery and time period were tested with an independent sample t-test, Pearson’s chi-square test, or Fisher’s exact test as appropriate for the data. Analysis was conducted with SAS v9.4 [21].

## Results

Of the 226 completed surveys, 121 were from knee and 105 were hip replacement patients (Table 1). Respondents were ages 35 to 86 (M = 65), predominately non-Hispanic White (95%), female (60%), and married or living with a partner (69%). The majority of participants were retired or employed for wages and had completed college or a higher degree. There were no significant differences in demographic attributes between hip and knee joint replacement patients. The majority of patients rated their self-perceived health as excellent or very good.

**Table 1:** Participant demographics

	Total *n* = 226	Knee replacement *n* = 121	Hip replacement *n* = 105	*p*
Female	135 (59.7)	76 (62.8)	59 (56.2)	0.31
Age, mean (SD), range	65.2 (10.2) 35–86	65.8 (9.5) 35–85	64.5 (10.8) 38–86	0.31
Race				0.36
Native American	1 (0.5)	0 (0.0)	1 (1.0)	
Asian	1 (0.5)	0 (0.0)	1 (1.0)	
Black	7 (3.1)	5 (4.2)	2 (1.9)	
White	214 (96.0)	113 (95.8)	101 (96.2)	
Hispanic ethnicity	1 (0.5)	1 (0.9)	0 (0.0)	0.99
Marital status				0.39
Married/partner	157 (69.5)	87 (71.9)	70 (66.7)	
Divorced/widowed/single	69 (30.5)	34 (28.1)	35 (33.3)	
Employment				0.47
Employed for wages/self emp.	83 (36.9)	40 (33.3)	43 (41.0)	
Retired or homemaker	115 (51.1)	64 (53.3)	51 (48.6)	
Not working (+ unable, disability)	27 (12.0)	16 (13.3)	11 (10.5)	
Education				0.99
HS/graduated/GED	34 (15.0)	18 (14.9)	16 (15.2)	
Some college	69 (30.5)	36 (29.8)	33 (31.4)	
College graduate	73 (32.3)	40 (33.1)	33 (31.4)	
Graduate school	50 (22.1)	27 (22.3)	23 (21.9)	
Rating of overall health				0.63
Excellent	18 (8.0)	7 (5.8)	11 (10.6)	
Very good	95 (42.4)	52 (43.3)	43 (41.4)	
Good	88 (39.3)	48 (40.0)	40 (38.5)	
Fair/poor	23 (10.3)	13 (10.8)	10 (9.6)	

Values are expressed as count (%) unless otherwise specified

*p* value from Pearson chi-square test, Fisher’s exact test or independent samples t-test

Respondents first selected their top three most important personal outcomes from the list of 17 potential outcomes (Table 2). The top-two most frequently selected outcomes were the same for both knee and hip replacement patients, ability to walk without pain/discomfort and pain relief, although their order of importance was reversed between groups. Both groups identified the ability to return to an active lifestyle (e.g., exercise, swimming, hiking) as the third most preferred outcome.

**Table 2:** Most important personalized outcomes for surgery in frequency order

	Total *n* = 224	Knee replacement *n* = 119	Hip replacement *n* = 105	*p*
Ability to walk without pain/discomfort	127 (56.7)	70 (58.8%)	57 (54.3%)	0.50
Pain relief	114 (50.9)	52 (43.7%)	62 (59.1%)	0.02
Ability to return to an active lifestyle (e.g., exercise, swimming, hiking)	80 (35.7)	40 (33.6%)	40 (38.1%)	0.49
Ability to return to leisure time activities (e.g., hobbies, gardening, games)	51 (22.8)	27 (22.7%)	24 (22.9%)	0.99
Ability to go up and down stairs without pain	48 (21.4)	35 (29.4%)	13 (12.4%)	0.002
Ability to sleep through the night	34 (15.2)	14 (11.8%)	20 (19.1%)	0.15
Improved ability to do work around my home	34 (15.2)	17 (14.3%)	17 (16.2%)	0.71
Improved ability to care for myself (dressing, getting socks/shoes on)	30 (13.4)	15 (12.6%)	15 (14.3%)	0.84
Increased strength	23 (10.3)	14 (11.8%)	9 (8.6%)	0.51
Improved flexibility	21 (9.4)	11 (9.2%)	10 (9.5%)	0.99
Ability to return to favorite sports activities (e.g. golf, softball)	21 (9.4)	11 (9.2%)	10 (9.5%)	0.99
Ability to return to work	20 (8.9)	13 (10.9%)	7 (6.7%)	0.35
Decreased numbness or weakness in my legs	8 (3.6)	5 (4.2%)	3 (2.9%)	0.73
Ability to sit comfortably	8 (3.6)	2 (1.7%)	6 (5.7%)	0.15
Improved relationships	5 (2.2)	3 (2.5%)	2 (1.9%)	0.99
Improved mental health	5 (2.2)	3 (2.5%)	2 (1.9%)	0.99
Ability to drive a car	4 (1.8)	3 (2.5%)	1 (1.0%)	0.62

Cell entries are the percentage of patients selecting an outcome as one of their three most important outcomes. Item order is based on item endorsement percentages from the pooled knee and hip data

*p* value from Fisher’s exact test

Other outcomes identified as important to patients included returning to leisure activities (hobbies, gardening, games), ability to go up and down stairs without pain, ability to sleep through the night, being able to work around their homes, and care for themselves. Pain relief was more important to hip patients (*p* = 0.02). The ability to go up and down stairs without pain was more important to knee patients (*p* = 0.002). Minor differences were noted by time period within surgery groups on returning to leisure activities, which was more important to hip replacement patients at 3-months than at pre-op or 12-months post-op (*p* < 0.05) and the ability to care for oneself for knee replacement patients (which increased in importance from pre-op through 3-and 12-months post-op, (*p* < 0.05).

We also asked patients whether they had discussed their important outcomes with their surgeon (Table 3). A majority of knee (62%) and hip patients (64%) said that it was very important that their surgeon know their preferred outcomes. Among those patients 75% said they had “definitely” discussed this with their surgeon. In contrast, among patients saying it was only somewhat or not important, only 23% reported definitely discussing this with their surgeon (*p* < 0.0001). There were no differences between hip and knee replacement patients in these two items, or by survey timing within each surgical group. The majority of post-operative patients (*n* = 151) perceived their surgery as successful, although there were significant differences by group with a smaller percentage of knee replacement patients identifying their surgery as definitely successful (*p* = 0.03). There were also significant differences in whether patients would opt to do their surgery over again, with a smaller percentage of knee surgery patients indicating they would compared to hip patients (*p* = 0.003). A smaller percentage at 12 months compared to 3 months indicated they would do the surgery again (*p* = 0.02). When asked about the degree to which they had achieved their personal outcomes from surgery, far fewer patients overall (35%) responded they had completely done so, with most patients indicating they had mostly or partially achieved them. Here, there were significant differences between knee and hip replacement patients (*p* = 0.02) with a smaller percentage of knee replacement patients indicating success, with no differences within group by time.

**Table 3:** Personalized outcomes and perceptions of surgery success

	Total *n* = 226	Knee replacement *n* = 121	Hip replacement *n* = 105	*p*
Important that my surgeon knows my personal outcomes				0.67
Very important	143 (63.3)	75 (62.0)	68 (64.8)	
Somewhat or not at all important	83 (36.7)	46 (38.0)	37 (35.2)	
Discussed personal outcomes with surgeon				0.89
Yes, definitely	126 (56.3)	68 (56.7)	58 (55.8)	
Yes, somewhat/no	98 (43.8)	52 (43.4)	46 (45.3)	
Surgery was a success (n = 151 post-surgery responses only)				0.03
Yes, definitely	114 (76.0)	56 (69.1)	58 (84.1)	
Yes, somewhat/no	36 (24.0)	25 (30.9)	11 (15.9)	
Would do surgery over again (n = 151 post-surgery responses only)				0.003
Yes, definitely	125 (82.8)	61 (74.4)	64 (92.8)	
Yes, somewhat/no	26 (17.2)	21 (25.6)	5 (7.2)	
To what degree achieved outcomes hoped for from surgery (n = 151 post-surgery responses only)				0.02
Completely	53 (35.1)	21 (25.6)	32 (46.4)	
Mostly	72 (47.7)	46 (56.1)	26 (37.7)	
Partially, a little, not at all	26 (17.2)	15 (18.3)	11 (15.9)	

*p* value from Pearson chi-square test

In considering how patients think about their outcomes from surgery, we also asked about their perceptions regarding the use of PROMs in their care. Summary scores from PROMs, (Oxford Knee or Oxford Hip Score, and PROMIS 10 Score) are provided to orthopedic patients upon completion of the PROMs survey before and after surgery. Only a minority of patients (42%) perceived these scores as very useful (Table 4). When asked about whether tracking a personal outcome they chose would be useful to them, the majority of patients (54%) perceived this as very useful. When asked whether they would prefer a PROM score or personalized outcome they identified, more than twice as many selected the personalized outcome (57%) as the PROM score (26%). The majority of patients (57%) also preferred that their surgeon use a personalized outcome rather than a summary score to assess progress and guide recovery over time, and thought it was more important (60%) that their surgeon knew their personal outcome rather than their PROM score. There were no differences between groups or within groups by time in relation to surgery on these questions.

**Table 4:** Perceptions of personalized outcomes, standardized PROMs and shared decision making

	Total *n* = 226	Knee replacement *n* = 121	Hip replacement *n* = 105	*p*
Usefulness of a summary PROM score for you and your care				0.44
Very useful	94 (41.8)	53 (44.2)	41 (39.1)	
Somewhat or not at all useful	131 (58.2)	67 (55.8)	64 (61.0)	
Usefulness of tracking progress of a personal outcome you chose as important				0.69
Very useful	121 (53.8)	66 (55.0)	55 (52.4)	
Somewhat or not at all useful	104 (46.2)	54 (45.0)	50 (47.6)	
Prefer your surgeon has a summary score or personalized outcome you chose				0.58
Summary score	58 (25.9)	34 (28.6)	24 (22.9)	
Important outcomes I chose	127 (56.7)	66 (55.5)	61 (58.1)	
Does not matter	39 (17.4)	19 (16.0)	20 (19.1)	
Important for surgeon to know your personalized outcome or general outcome				0.11
Personalized outcome	136 (60.2)	80 (66.1)	56 (53.3)	
General outcome	76 (33.6)	36 (29.8)	40 (38.1)	
Does not matter to me	14 (6.2)	5 (4.1)	9 (8.6)	
Discussed other important factors in life with surgeons or care team				0.20
Yes	152 (76.0)	82 (75.2)	70 (76.9)	
No, information is available in my medical record	23 (11.5)	16 (14.7)	7 (7.7)	
No, I do not think information is important for them to know	25 (12.5)	11 (10.9)	14 (15.4)	
Importance of shared decision-making for care after surgery				0.23
Very important	178 (78.8)	99 (81.8)	79 (75.2)	
Somewhat or not important	48 (21.2)	22 (18.2)	26 (24.8)	

Values are expressed as count (%) unless otherwise specified

*p* values from Pearson chi-square test

The items that patients identified as important factors in their lives that could affect their care following surgery are presented in Table 5. Significant concerns noted by 20% or more of respondents addressed a range of issues including infection, pain medication, navigating stairs, handicap accessibility, living alone, and caring for their pets. There were no statistical differences between groups regarding the selection of items important to them. The majority of patients (76%) said they had discussed these factors with their surgeons. The majority of patients (79%) also said that it was very important for them to share in the decision-making process about their care after surgery.

**Table 5:** Issues affecting patients post-surgery

	Knee replacement *n* = 121	Knee replacement *n* = 121	Hip replacement *n* = 105	*p*
I have stairs inside my home	139 (61.5)	77 (63.6)	62 (59.1)	0.50
I have to climb stairs to get into my house	97 (42.9)	50 (41.3)	47 (44.8)	0.69
I’m concerned about infections	70 (31.0)	40 (33.1)	30 (28.6)	0.48
My home is not handicapped accessible	64 (28.3)	37 (30.6)	27 (25.7)	0.46
I’m concerned about pain medications	57 (25.2)	31 (25.6)	26 (24.8)	0.99
I live alone	45 (19.9)	25 (20.7)	20 (19.1)	0.87
I have pets to care for	45 (19.9)	21 (17.4)	24 (22.9)	0.32
My family and friends are not available to help me	23 (10.2)	10 (8.3)	13 (12.4)	0.38
I get nauseated from anesthesia	22 (9.7)	11 (9.1)	11 (10.5)	0.82
I must use stairs to get to the bathroom	21 (9.3)	10 (8.3)	11 (10.5)	0.65
I’m concerned about affording follow-up care	18 (8.0)	9 (7.4)	9 (8.6)	0.81
I am a primary caregiver for someone else	10 (4.4)	3 (2.5)	7 (6.7)	0.19
I have young children to care for	8 (3.5)	4 (3.3)	4 (3.8)	0.99
I cannot swallow large pills	8 (3.5)	4 (3.3)	4 (3.8)	0.99
I fear needles	7 (3.1)	5 (4.1)	2 (1.9)	0.45
I don’t have transportation to get to the doctor or physical therapy	6 (2.7)	4 (3.3)	2 (1.9)	0.69

Items ordered by endorsement in the pooled knee and hip sample

*p* value from Fisher’s exact test

## Discussion

Patients scheduled for or having recently had knee or hip replacement surgery clearly identified personal outcomes they want from their surgery that are important and meaningful to them. While some of these outcomes, such as physical functioning and pain relief, align with items within various PROM measures, there were a variety of other outcomes identified that are not usually included in PROMs for orthopedic care, such as specific aspects of returning to an active lifestyle or engaging in specific leisure activities. Without knowing those specifics, it will be difficult for care teams to formulate postoperative programs to achieve them (e.g., rehabilitation needed for hiking versus playing soccer). Patients also indicated they preferred a personal outcome that they identified to track progress in their care rather than an overall PROM score and thought it very important their surgeon know their desired personal outcomes. The majority of patients also clearly indicated a desire to engage in shared decision-making about their post-surgical care and treatment, with patients identifying personal factors about their lives that could affect their care, such as living alone, and caring for pets—which may not always be discussed in the context of aftercare planning. Complementing the use of PROMs scores with the addition of assessing the attainment of a personal outcome chosen by the patient could add value to PROMs in clinical care, encouraging patients to reflect on what they want from their surgery and facilitating more robust patient communication and involvement in shared decision-making especially for post-operative care and treatment. This may also increase patient satisfaction with care and its outcomes.

Personal outcomes identified by patients are by nature unique and tailored to an individual patient’s life and perspective. Standard outcomes assumed important to patients such as pain relief or walking without pain/discomfort may be a patient’s desired outcome, but these patients also seemed to think of outcomes in relation to a specific activity of daily life, or a specific sport or leisure activity that is meaningful and important to them. While these activities may indeed require improvement in pain or functional status, those symptoms may not necessarily be the primary focus of the patient. The use of PROMs in clinical care was not designed to identify or track patient desired outcomes, but rather focus on assessing or tracking symptoms, severity, and functional status, factors which are modifiable in a clinical context. While a patient’s personal outcomes, such as returning to an active lifestyle or re-engaging in specific sport or leisure activity, may not appear to have a clear clinical context, clarifying how these activities relate to outcomes that can be addressed in a clinical context, such as pain relief, symptom severity and functional status, could help patients more clearly understand the relationship of PROMs to their own personal outcomes, tying them more clearly together.

While there has been research in orthopedics on what patients expect or prefer in their care, expectations and preferences for overall care hold differing meaning than what patients desire as outcomes from surgical care. Wright et al. for instance developed the Knee Patient Specific Index (KPSI), a PROM designed to provide more individualized measurement of the type, severity, and importance of patient complaints in orthopedic care [19, 22]. This PROM however, like many other highly regarded PROMs for orthopedics, is based on a volume of questions, 22 in this case, that form a summary numbered score. It takes time and effort to complete, and the resulting score may not be understood well by the patient. Patient preferences for personal outcomes could be the result of a lack of knowledge on how to use and interpret PROMs in the context of their personal goals and outcomes from surgery. There is currently little education for patients about the purpose and use of PROMs. In the qualitative study this research was based on, patients who were interviewed about PROMs frequently did not remember receiving their scores or thought the score was for the surgeon’s use, not theirs with many stating that they did not understand what the PROMs score meant [18]. This may be why most patients in this study identified their personal outcomes as more useful and important to them than PROMs scores and wanted their surgeons to know about those outcomes and use them for assessing progress in their care. For a PROM score to be useful and meaningful to patients they would need to understand what the score reflects about their functioning and its meaning in relation to their desired care outcome. This would entail much more robust communication and education on PROMs, including how they are used in clinical decision-making. Field and colleagues [23] in addressing the use of PROMs in clinical care, note that PROMs can be a tool for facilitating and enhancing communication and decision-making, benefiting the patient–clinician encounter and helping manage patient expectations for their care. Adding a personalized outcome in addition to PROMs could facilitate this communication even more, engaging the patient in their desired outcome while explaining and weaving in their PROMs score and how it relates to their desired outcome. It may also encourage PROM completion by patients, currently a challenging endeavor in most health systems [9, 24].

Post-surgical care is also an area important in patient care and communication. Here patients in the survey listed many factors that they perceived as important to their aftercare. While some of these factors would likely be discussed, there are others that are unique to a individuals life situation, such as living alone or caring for other people or pets, that may not arise in aftercare planning discussions. Care for companion animals for example, a growing phenomenon in U.S. households, is particularly unlikely to come up in a clinical discussion [25]. Facilitating communication that addresses the unique factors in a patient’s life is important to successful after-care. Fortunately, the majority of these patients wanted to be involved in shared decision-making for their care following surgery, providing an opportunity for robust discussion about the unique contextual factors in their lives that could affect their aftercare and treatment.

There are limitations to the current study. The survey response rate of 56% has potential for selection bias and respondents also showed higher levels of education than the general population. The study was also conducted at a single site and the narrow patient diversity and lack of longitudinal data limit generalizability. Patients in this study were also familiar with filling out PROMs for their care, a concept that has yet to have wide-spread use across health care. The study also has strengths however, building as it did upon initial interviews with patients and thus assuring their voice was well-represented in survey development and spoke to what was meaningful to them. We also note the study had patient engagement and representation from study design through completion, including a patient-investigator [CN] on the study team to bring this important perspective to the conduct of the research. The study also involved patients at different time points in the surgical trajectory, that began prior to and extended to one-year post-surgery, exploring varying perceptions at different timepoints among patients.

## Conclusions

The inclusion of PROMs in clinical care has arrived with both promise and challenge in the care of patients. The promise is of additional clinical tools for decision-making, monitoring, and potentially enhancing patient involvement in their care. This however will require addressing the challenge of engaging and educating patients, and also likely clinicians, about PROMS and how they can benefit patient care, for while patients continue to be involved in development studies for PROMs, many, including patients in this study, are still not seeing their personal outcomes represented in the PROMs used. Adding a personal outcome to PROMs that is meaningful and important to a patient may be a bridge in that divide, aligning patient treatment and outcomes with patient priorities and what patients perceive as most meaningful in their care. Involving patients in their care is a key to good clinical outcomes, as well as patient satisfaction, and is at the heart of transforming healthcare to be more patient-centered.

## Data Availability

The datasets generated during and/or analyzed during the current study are not publicly available but are available upon reasonable request.

## References

[1] Lowry KJ, Brox WT, Naas PL, Tubb CC, Muschler GF, Dunn W (2019) Musculoskeletal-based patient-reported outcome performance measures, where have we been—where are we going. J Am Acad Orthop Surg 27(13):e589–e595. 10.5435/jaaos-d-18-00429 31232793 10.5435/JAAOS-D-18-00429

[2] Siljander MP, McQuivey KS, Fahs AM, Galasso LA, Serdahely KJ, Karadsheh MS (2018) Current trends in patient-reported outcome measures in total joint arthroplasty: a study of 4 major orthopaedic journals. J Arthroplasty 33(11):3416–3421. 10.1016/j.arth.2018.06.034 30057269 10.1016/j.arth.2018.06.034

[3] Gagnier JJ (2017) Patient reported outcomes in orthopaedics. J Orthop Res 35(10):2098–2108. 10.1002/jor.23604 28513993 10.1002/jor.23604

[4] MOTION Group (2018) Patient-reported outcomes in orthopaedics. J Bone Jt Surg Am 100(5):436–442. 10.2106/jbjs.17.00608 10.2106/JBJS.17.00608 29509622

[5] Lavallee DC, Chenok KE, Love RM, Petersen C, Holve E, Segal CD et al (2016) Incorporating patient-reported outcomes into health care to engage patients and enhance care. Health Affairs (Project Hope) 35(4):575–582. 10.1377/hlthaff.2015.1362 27044954 10.1377/hlthaff.2015.1362

[6] Camuso N, Bajaj P, Dudgeon D, Mitera G (2016) Engaging Patients as Partners in developing patient-reported outcome measures in cancer—a review of the literature. Support Care Cancer 24(8):3543–3549. 10.1007/s00520-016-3151-0 27021391 10.1007/s00520-016-3151-0

[7] Porter I, Gonçalves-Bradley D, Ricci-Cabello I, Gibbons C, Gangannagaripalli J, Fitzpatrick R et al (2016) Framework and guidance for implementing patient-reported outcomes in clinical practice: evidence, challenges and opportunities. J Comp Eff Res 5(5):507–519. 10.2217/cer-2015-0014 27427277 10.2217/cer-2015-0014

[8] Ayers DC, Zheng H, Franklin PD (2013) Integrating patient-reported outcomes into orthopaedic clinical practice: proof of concept from FORCE-TJR. Clin Orthop Relat Res 471(11):3419–3425. 10.1007/s11999-013-3143-z 23925525 10.1007/s11999-013-3143-z PMC3792269

[9] Bilimoria KY, Cella D, Butt Z (2014) Current challenges in using patient-reported outcomes for surgical care and performance measurement: everybody wants to hear from the patient, but are we ready to listen? JAMA Surg 149(6):505–506. 10.1001/jamasurg.2013.5285 24872165 10.1001/jamasurg.2013.5285

[10] Gagnier JJ, Mullins M, Huang H, Marinac-Dabic D, Ghambaryan A, Eloff B et al (2017) A systematic review of measurement properties of patient-reported outcome measures used in patients undergoing total knee arthroplasty. J Arthroplasty 32(5):1688–97.e7. 10.1016/j.arth.2016.12.052 28162839 10.1016/j.arth.2016.12.052

[11] McKenna SP (2011) Measuring patient-reported outcomes: moving beyond misplaced common sense to hard science. BMC Med 9:86. 10.1186/1741-7015-9-86 21756344 10.1186/1741-7015-9-86 PMC3170214

[12] Wiering B, de Boer D, Delnoij D (2017) Patient involvement in the development of patient-reported outcome measures: a scoping review. Health Expect Int J Public Particip Health Care Health Policy 20(1):11–23. 10.1111/hex.12442 10.1111/hex.12442 PMC5217930 26889874

[13] Zura R, Steen RG (2018) Patient-reported outcome measures in perspective. Orthopedics 41(1):10–11. 10.3928/01477447-20180109-03 29401367 10.3928/01477447-20180109-03

[14] Wiering B, de Boer D, Delnoij D (2017) Patient involvement in the development of patient-reported outcome measures: the developers’ perspective. BMC Health Serv Res 17(1):635. 10.1186/s12913-017-2582-8 28886742 10.1186/s12913-017-2582-8 PMC5591531

[15] Wiering B, de Boer D, Delnoij D (2017) Asking what matters: the relevance and use of patient-reported outcome measures that were developed without patient involvement. Health Expect Int J Public Particip Health Care Health Policy 20(6):1330–1341. 10.1111/hex.12573 10.1111/hex.12573 PMC5689236 28675514

[16] Marshall S, Haywood K, Fitzpatrick R (2006) Impact of patient-reported outcome measures on routine practice: a structured review. J Eval Clin Pract 12(5):559–568. 10.1111/j.1365-2753.2006.00650.x 16987118 10.1111/j.1365-2753.2006.00650.x

[17] Boyce MB, Browne JP, Greenhalgh J (2014) The experiences of professionals with using information from patient-reported outcome measures to improve the quality of healthcare: a systematic review of qualitative research. BMJ Qual Saf 23(6):508–518. 10.1136/bmjqs-2013-002524 10.1136/bmjqs-2013-002524 24505110

[18] Whitebird RR, Solberg LI, Norton CK, Ziegenfuss JY, Asche SE, Grossman ES (2020) What outcomes matter to patients after joint or spine surgery? J Patient Cent Res Rev 7(2):157–164. 10.17294/2330-0698.1738 32377549 10.17294/2330-0698.1738 PMC7197885

[19] Wright JG, Santaguida PL, Young N, Hawker GA, Schemitsch E, Owen JL (2010) Patient preferences before and after total knee arthroplasty. J Clin Epidemiol 63(7):774–782. 10.1016/j.jclinepi.2009.08.022 20004554 10.1016/j.jclinepi.2009.08.022

[20] Naik AD, Catic A (2020) Achieving patient priorities: an alternative to patient-reported outcome measures (PROMs) for promoting patient-centered care. BMJ Qual Saf. 10.1136/bmjqs-2020-012244 33115850 10.1136/bmjqs-2020-012244

[21] SAS Institute. SAS V9.4. Cary NC

[22] Wright JG, Young NL (1997) The patient-specific index: asking patients what they want. J Bone Jt Surg Am 79(7):974–983. 10.2106/00004623-199707000-00003 10.2106/00004623-199707000-00003 9234873

[23] Field J, Holmes MM, Newell D (2019) PROMs data: can it be used to make decisions for individual patients? A narrative review. Patient Relat Outcome Meas 10:233–241. 10.2147/prom.S156291 31534379 10.2147/PROM.S156291 PMC6681163

[24] Slover JD, Karia RJ, Hauer C, Gelber Z, Band PA, Graham J (2015) Feasibility of integrating standardized patient-reported outcomes in orthopedic care. Am J Manag Care 21(8):e494-500 26625504

[25] American Veterinary Medical Association. AVMA U.S. Pet Ownership & Demographics Sourcebook. 2017–2018 edition

